# Causes contributing to the excess maternal mortality risk for women 35 and over, United States, 2016–2017

**DOI:** 10.1371/journal.pone.0253920

**Published:** 2021-06-29

**Authors:** Marian F. MacDorman, Marie Thoma, Eugene Declercq, Elizabeth A. Howell

**Affiliations:** 1 Maryland Population Research Center, University of Maryland, College Park, MD, United States of America; 2 Department of Family Science, University of Maryland School of Public Health, College Park, MD, United States of America; 3 Department of Community Health Sciences, Boston University School of Public Health, Boston, MA, United States of America; 4 Department of Obstetrics and Gynecology, University of Pennsylvania, Philadelphia, PA, United States of America; Universita degli Studi di Milano-Bicocca Scuola di Medicina e Chirurgia, ITALY

## Abstract

To better understand age-related disparities in US maternal mortality, we analyzed 2016–2017 vital statistics mortality data with cause-of-death literal text (actual words written on the death certificate) added. We created a subset of *confirmed* maternal deaths which had pregnancy mentions in the cause-of-death literals. Primary cause of death was identified and recoded using cause-of-death literals. Age-related disparities were examined both overall and by primary cause. Compared to women <35, the 2016–2017 US maternal mortality rate was twice as high for women aged 35–39, four times higher for women aged 40–44, and 11 times higher for women aged 45–54 years. Obstetric hemorrhage was the leading cause of death for women aged 35+ with rates 4 times higher than for women <35, followed by postpartum cardiomyopathy with a 3-fold greater risk. Obstetric embolism, eclampsia/preeclampsia, and Other complications of obstetric surgery and procedures each had a two-fold greater risk of death for women aged 35+. Together these 5 causes of death accounted for 70.9% of the elevated maternal mortality risk for women aged 35+. The excess maternal mortality risk for women aged 35+ was focused among a few causes of death and much of this excess mortality is preventable. Early detection and treatment, as well as continued care during the postpartum year is critical to preventing these deaths. The Alliance for Innovation on Maternal Health has promulgated patient safety bundles with specific interventions that health care systems can adopt in an effort to prevent these deaths.

## Introduction

Researchers have long noted an increased risk of maternal death for women aged 35+ [1–3], as well as higher risks for conditions associated with severe morbidity and mortality, including sepsis [4], venous thromboembolism [5] and hypertensive disorders [6]. However, characterization of this increased risk has been complicated by concerns about the accuracy of US vital statistics maternal mortality data [7, 8]. A pregnancy checkbox was added to the death certificate to improve reporting of maternal deaths [7, 8]. However, recent validity studies found that errors in the pregnancy checkbox led to overreporting of maternal deaths, ranging from 21% in a 4 state study [7] to 50% in a Texas study [8]. The degree of misclassification increased with maternal age with the greatest misclassification among decedents aged 40+ [9, 10]. In addition, 40–50% of maternal deaths were coded to ill-defined causes that provide virtually no information as to the actual cause of death [11, 12]. We applied alternative coding methods to create a set of confirmed maternal deaths with increased specificity of cause-of-death coding [11, 12]. The purpose of this study was to use this set of confirmed maternal deaths to re-examine age-related disparities in US maternal mortality, to identify the leading causes of maternal death by age, and to identify the specific causes of death that contributed the most to age-related disparities.

## Methods

We used the 2016–2017 U.S. vital statistics multiple-cause-of-death data files produced by the National Center for Health Statistics (NCHS), with cause-of-death literals added. The cause-of-death literals are the actual words written in the cause-of-death section of the death certificate, which provide much richer detail about the actual circumstances of death than the ICD-10 codes. Maternal deaths (International Classification of Diseases, Tenth Revision (ICD-10) codes A34, O00-O95, O98-O99) are defined as the death of a woman while pregnant or within 42 days of the end of pregnancy from any cause related to or aggravated by the pregnancy or its management, but not from accidental or incidental causes. Late maternal deaths (ICD-10 code 096) are those that occur from 43 days-1 year after pregnancy [13].

We created a subset of all possible maternal or late maternal deaths for coding. This included all females aged 10–54 with a maternal or late maternal code in the multiple cause-of-death data OR with pregnant/postpartum status indicated by the pregnancy checkbox [11, 12]. We developed an alternative coding strategy to identify the *primary* cause of death directly from the cause-of-death literals using methods described in more detail elsewhere [11, 12]. Standard procedures for coding rely heavily on the sequence in which causes are written on the death certificate; however, sometimes the sequence is inconsistent. Instead, we defined the “primary cause of death” as the most likely, or primary, cause that led to the decedent’s death, regardless of order of terms listed on the death certificate [11, 12]. Due to major problems with the pregnancy checkbox data [7, 8], we recoded records with a pregnancy/postpartum mention in the cause-of-death literals to maternal causes, and records with no such mention to non-maternal causes [11, 12]. Late maternal deaths with pregnancy mentions were recoded to more specific primary maternal causes.

Our population of maternal deaths included those deaths that could be **confirmed** as maternal from specific terms listed in the cause-of-death literals. Our maternal mortality rates likely underestimate the true levels of maternal death in the United States if the woman’s pregnancy status was not mentioned in the literals. Thus, we focused on relative associations (i.e., rate ratios, percent contribution) across age groups. The advantages of this approach were to: 1) identify a set of deaths that we can clearly confirm as maternal deaths 2) to improve the specificity of cause-of-death coding for these deaths, and 3) to greatly reduce the percentage of deaths coded to ill-defined causes (from 43.5% in the NCHS-coded data to 2.5% among confirmed maternal deaths) [11].

Maternal mortality rates were computed per 100,000 live births. Maternal mortality rate ratios were computed as the maternal mortality rate for group A (women aged 35+) divided by the maternal mortality rate for group B (women <35). Text statements were tested for statistical significance and a statement that a rate is higher or lower than another rate indicates that the rates were significantly different at the p < .05 level. The study was ruled exempt from IRB review by the University of Maryland IRB as the study was based on death certificates and there were no living human subjects.

## Results

**Fig 1:**
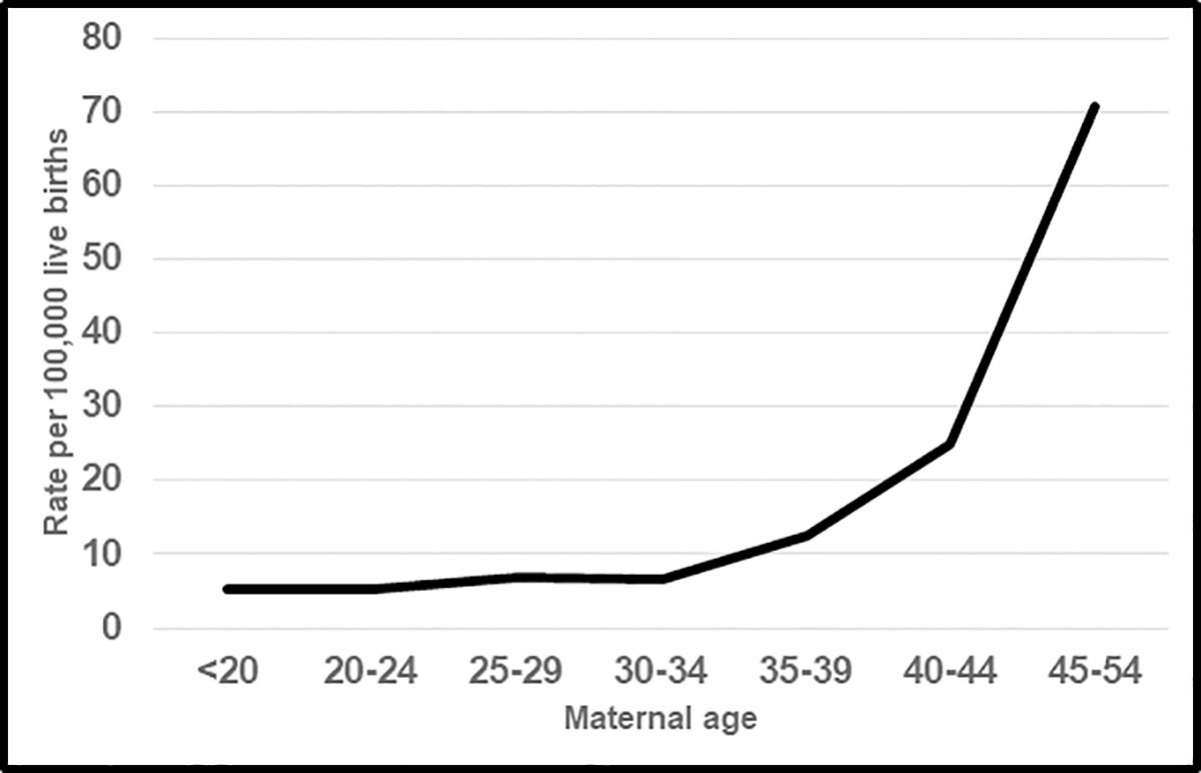
Confirmed maternal mortality rates by maternal age, United States, 2016–2017.

Compared to women <35 years of age, the confirmed maternal mortality rate was twice as high for women aged 35–39 (mortality rate ratio (MRR) = 1.98), four times higher for women aged 40–44 (MRR = 3.96), and 11 times higher for women aged 45–54 years (MRR = 11.22) (Fig 1).

For the total population, obstetric embolism and eclampsia/preeclampsia were tied for the leading cause of maternal death (Table 1). The obstetric embolism category includes amniotic fluid, pulmonary, and any other embolism occurring during the pregnant/postpartum period. The third leading cause of death was postpartum cardiomyopathy, followed by obstetric hemorrhage, and other complications of obstetric surgery and procedures. About 2/3 (65.7%) of confirmed maternal deaths were due to one of these five causes.

**Table 1:** Five leading causes of confirmed maternal death by maternal age, United States, 2016–2017.

	Total^1^			Maternal age <35			Maternal age 35+			Rate ratio
Primary cause of death (ICD-10 code)	Rank	Number	Rate	Rank	Number	Rate	Rank	Number	Rate	35+/<35
**Total**		615	7.88		407	6.31		208	15.42	2.45
Eclampsia and pre-eclampsia (O11, O13-O16)	1	98	1.26	1	72	1.12	4	26	1.93	1.73
Obstetric embolism (O88)	1	98	1.26	2	67	1.04	3	31	2.30	2.21
Postpartum cardiomyopathy (O90.3)	3	86	1.10	3	52	0.81	2	34	2.52	3.13
Obstetric hemorrhage (O20, O43.2, O44-O46, O67, O71.0, O71.1, O71.3, O71.4, O71.7, O72)	4	82	1.05	4	45	0.70	1	37	2.74	3.93
Other complications of obstetric surgery & procedures (O75.4)	5	40	0.51	5	26	0.40	5	14	1.04*	2.58

* Rate considered statistically unreliable; based on 10–19 deaths in the numerator.

Note: Maternal deaths include those during pregnancy and up to 42 days postpartum

Note: Denominators for rates are the number of live births in each group:: 7801375 Total; 6452772 <35; 1348603 35+.

**Fig 2:**
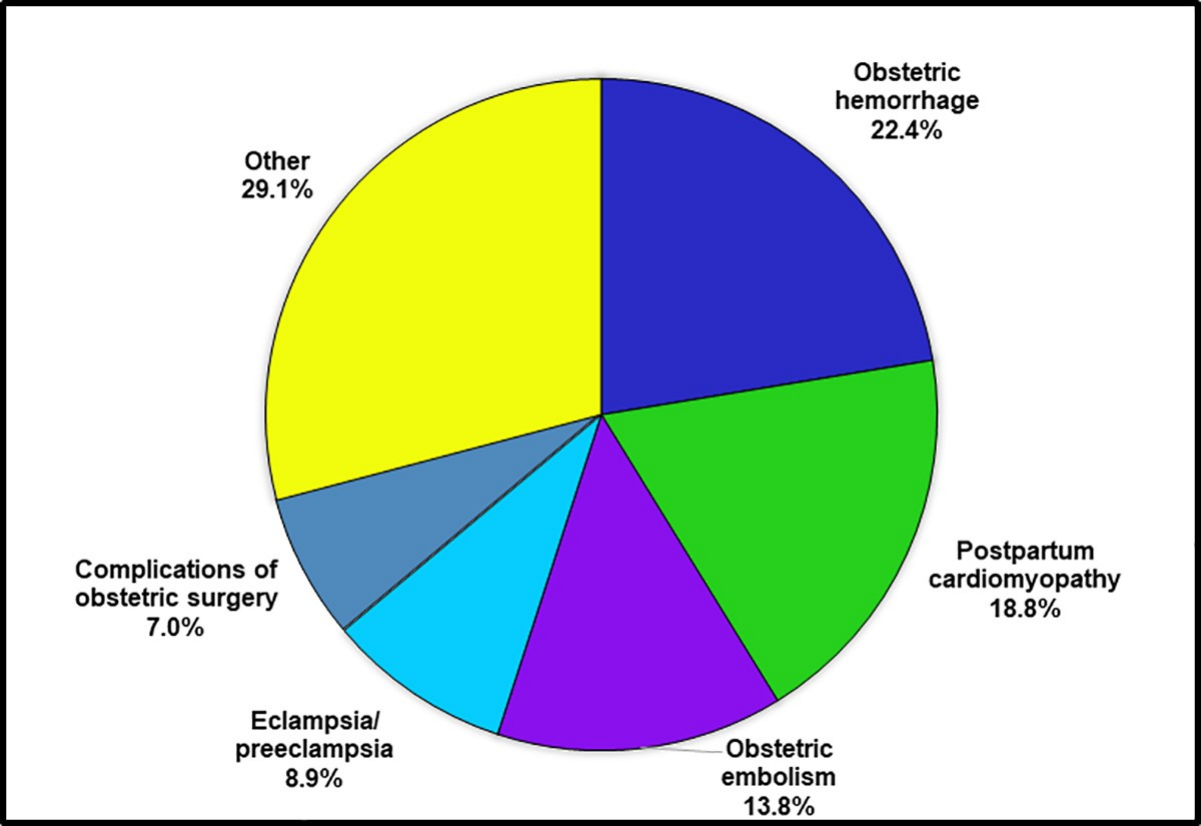
Percent contribution of leading causes of maternal death to the increased risk of maternal mortality for women age 35+, compared to women <35, United States, 2016–2017.

For women <35, the leading causes of death were similar to those for the total population. However, for women aged 35+, the leading causes were markedly different. Obstetric hemorrhage was the leading cause for women aged 35+, and the risk of death from obstetric hemorrhage was 4 times higher for women aged 35+ then for women <35 (MRR = 3.93). The second leading cause for women aged 35+ was postpartum cardiomyopathy, with a three times increased risk of death when compared to women <35 (MRR = 3.13). The third, fourth and fifth leading causes of death for women aged 35+ were obstetric embolism, eclampsia/preeclampsia, and other complications of obstetric surgery and procedures, with MRRs of 2.21, 1.73 and 2.58, respectively. The causes of death that contributed the most to the increased risk of maternal death for mothers 35+, compared to those less than 35, were obstetric hemorrhage (22.4%), postpartum cardiomyopathy (18.8%), and obstetric embolism (13.8%) (Fig 2).

**Fig 3:**
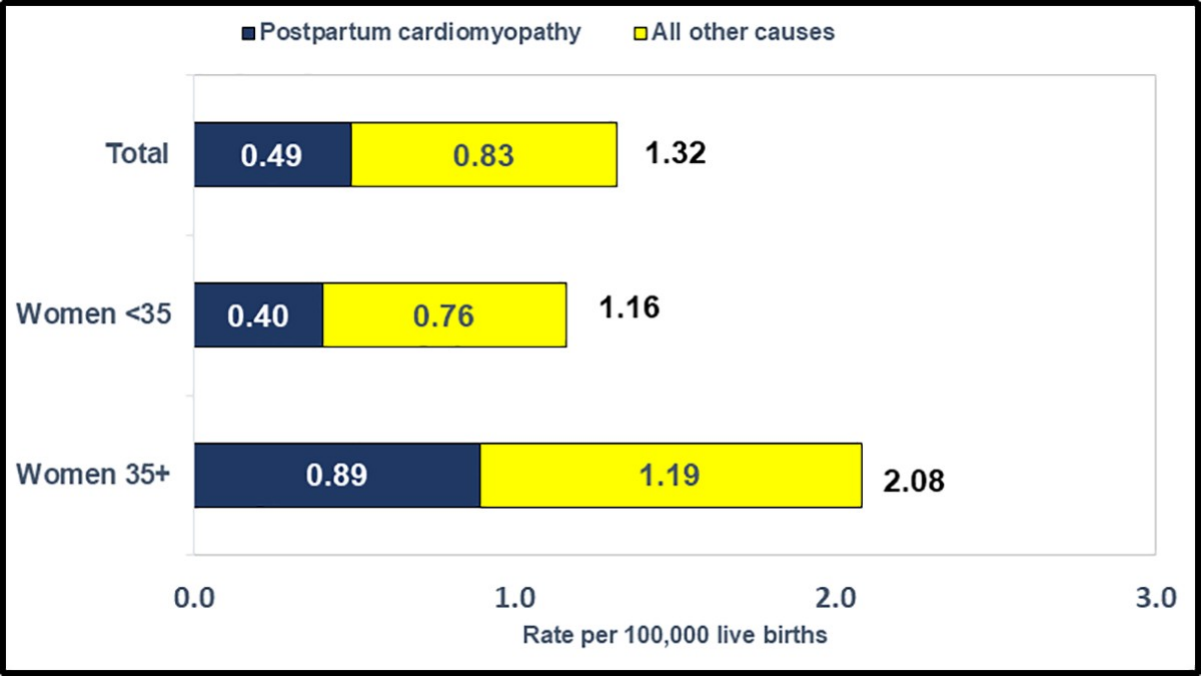
Late maternal mortality rates for postpartum cardiomyopathy and all other causes by maternal age, United States, 2016–2017.

Compared to women <35, late maternal mortality rates were markedly higher for women aged 35+ (MRR = 1.79) (Fig 3). For late maternal deaths, the leading cause of death was postpartum cardiomyopathy, and the postpartum cardiomyopathy risk was more than twice as high (MRR = 2.21) for women aged 35+ than for women <35. While postpartum cardiomyopathy accounted for 18.8% of the difference in maternal mortality risk between women 35+ and women <35, it accounted for over one-half (53.3%) of the total difference for late maternal mortality.

## Discussion

Maternal age increases the risk of maternal mortality, but examination of this relationship has been complicated by reporting errors which increased substantially with maternal age [9, 10]. Consistent with other studies [1–6], we found that maternal mortality risk increased progressively with increasing maternal age. Compared to women <35 years of age, the 2016–2017 US maternal mortality rate was twice as high for women aged 35–39, four times higher for women aged 40–44, and 11 times higher for women aged 45–54 years.

To reduce reporting errors due to the checkbox, NCHS changed coding of maternal deaths for data years 2018 onward to retain the use of the pregnancy checkbox for decedents aged 10–44 years, and to not use the checkbox for decedents aged 45+ [13]. This differential coding practice may obfuscate true differences in age-related risks. For example, in a 2020 report, the maternal mortality rate, albeit based on small numbers, was nonsignificantly lower for women aged 45–54 than for those aged 40–44 [13]. In contrast, our findings indicate higher risk among women aged 45–54 using a consistently constructed method across age groups.

We found that the excess risk of maternal mortality for women aged 35+ was concentrated among a few causes of death. Compared to women <35, the risk of maternal death from obstetric hemorrhage was 4 times higher, the risk of postpartum cardiomyopathy was 3 times higher, and the risk of obstetric embolism and eclampsia/preeclampsia were twice as high for women aged 35+. Studies have shown that up to 70% of hemorrhage deaths [14], 60% of eclampsia/preeclampsia deaths [15], and a significant proportion of both embolism [14] and cardiomyopathy [16] deaths are preventable.

### Strengths and limitations

This study used a set of confirmed maternal deaths with improved cause-of-death coding to more accurately assess the increased risk of maternal death for women aged 35+. Strengths of this study include the use of cause-of-death literals, which provide richer detail on the specific circumstances of death often lost during standard coding processes. For many women, an examination of the cause-of-death literals provided confirmation that the woman was pregnant or postpartum at the time of death, thus confirming the accuracy of the maternal death attribution. Examination of the literals together with improved coding procedures reduced the percentage of deaths coded to ill-defined causes from 43.5% in the NCHS-coded data to 2.5% among confirmed maternal deaths, demonstrating that most records initially coded to ill-defined causes actually contained more specific cause-of-death information, as previously reported [11].

A major limitation of the study is that some actual maternal deaths were likely not included in our subset of confirmed maternal deaths, if a woman’s pregnant/postpartum status was not mentioned in the cause-of-death literals. Because of this, we emphasized rate ratios and percent contribution among confirmed maternal deaths, rather than focusing on maternal mortality rates. It is also possible that the degree of underreporting may vary by cause if some causes of death were more or less likely to be accompanied by a pregnancy/postpartum mention in the cause-of-death literals. Thus, results may not be generalizable to all maternal deaths in the U.S.

## Conclusions

The excess maternal mortality risk for women aged 35+ was focused among a few causes of death, and much of this excess mortality is preventable [14–16]. The prominence of hemorrhage, cardiomyopathy, eclampsia/preeclampsia, and embolism among the leading causes of death for women aged 35+ highlights the urgent need for greater attention to these potentially preventable complications. Similar to work in other countries [17], The Alliance for Innovation on Maternal Health (AIM) has developed patient safety bundles with specific procedures that health care systems can implement in an effort to prevent deaths and severe morbidity from these causes [18]. These bundles include critical steps to improve readiness to respond to obstetric emergencies, training for early identification and prevention of complications, standardized response protocols to optimize treatment, and protocols for reporting and learning from adverse events. In addition, to prevent maternal deaths from these and other causes, increased vigilance is needed to promptly identify and address both social and medical risk factors across the pregnancy care continuum, including throughout the postpartum year [19, 20].
